# Acute Ischemic Stroke in Non-Arteritic Anterior Ischemic Optic Neuropathy

**DOI:** 10.3390/diagnostics15243192

**Published:** 2025-12-14

**Authors:** Victor Wenzel, Leon Alexander Danyel, Sophia Meidinger, Eberhard Siebert, Theresia Knoche, Charlotte Pietrock

**Affiliations:** 1 Department of Neurology with Experimental Neurology, Charité–Universitätsmedizin Berlin, Corporate Member of Freie Universität Berlin, Humboldt-Universität zu Berlin, and Berlin Institute of Health, Augustenburger Platz 1, 13353 Berlin, Germany; 2 Institute of Neuroradiology, Charité–Universitätsmedizin Berlin, Corporate Member of Freie Universität Berlin, Humboldt-Universität zu Berlin, and Berlin Institute of Health, Charitéplatz 1, 13353 Berlin, Germany

**Keywords:** optic neuropathy, NAION, magnetic resonance imaging, diffusion-weighted imaging, stroke

## Abstract

**Background:** Non-arteritic anterior ischemic optic neuropathy (NAION) is a neuroophthalmological disorder characterized by impaired blood flow to the optic nerve head. There is uncertainty about whether, in some cases, NAION may be caused by proximal embolism of the posterior ciliary arteries. Diffusion-weighted magnetic resonance imaging (DWI-MRI) can provide evidence of concurrent cerebral infarction that may indicate a common embolic etiology. **Methods:** Adults with ophthalmological diagnosis of NAION who underwent cerebral DWI-MRI within 14 days from onset of visual impairment were included in a retrospective cohort study (2013–2021). DWI-MRI images were assessed for presence, location, and type of ischemic stroke by a board-certified neuroradiologist blinded for clinical patient data. **Results:** Among 122 patients (mean age 64.6 ± 11.9 years), DWI-MRI indicated acute/subacute ischemic stroke in three cases (2.5%), all located within the anterior circulation in the territory of the left middle cerebral artery and ipsilateral to the affected eye in two cases (1.6%). Ischemic stroke location was cortical in one case (0.8%) and subcortical in two cases (1.6%). Acute ischemic stroke indicated by a hyperintense DWI signal and corresponding low ADC was present in one patient (0.8%). Two patients (1.6%) had subacute ischemic stroke (hyperintense DWI signal and normal or elevated ADC signal). Only one NAION patient (0.8%) had acute embolic stroke corresponding to the vascular territory of the affected eye. **Conclusions:** Concurrent embolic ischemic stroke in NAION is exceedingly rare. Our findings support the prevailing pathophysiological theory of NAION as a non-embolic disease.

## 1. Introduction

Ischemic optic neuropathies (ION) constitute one of the leading causes of visual impairment worldwide [1,2,3,4,5]. They are caused by a vascular insufficiency of the optic nerve and are classified into two types based on the region of the nerve affected, as follows: anterior ION (AION) involving the optic nerve head supplied by the short posterior ciliary arteries, and posterior ION (PION) for the rest of the optic nerve supplied by a complex system of branches of the central retinal artery and collateral arteries of the ophthalmic artery [1,2,3,4,6]. Of these, AION is far more common, representing about 90% of all ION cases [1,2,3,4,6]. It is further subdivided into two clinical types: non-arteritic (NAION) and arteritic (A-AION) AION [1,2,3,4].

NAION accounts for 90–94% of all AION cases, making it the most common ION [2,3]. The annual incidence rate increases with age and is estimated at 2.3–10.2 per 100,000 people [4,5,7,8,9,10]. NAION is generally considered a disease of the middle-aged and elderly; however, in 7.5–23% of cases, patients are younger than 50 years [5,9,11,12]. Although long believed to affect both sexes equally, recent studies suggest that NAION is more prevalent in males [5,13]. Clinically, NAION presents with painless monocular visual impairment, which can appear suddenly, upon awakening, or gradually deteriorate over days to weeks [1,2,4,5,14]. Patients experience relative or absolute visual field defects, which may be central, sectoral, altitudinal, or a combination of these, reflecting the underlying vascular pattern, which can vary considerably between patients [1,3,4]. Visual acuity is not necessarily affected and may range from normal vision to blindness [1,4]. Visual impairment is often accompanied by a relative afferent pupillary defect (RAPD). The fundoscopic examination reveals an edematous optic disc, which develops pallor over time [1,4]. There are no established diagnostic criteria of NAION to date: diagnosis is primarily clinical and based on a combination of symptomology, evidence of optic disc edema in fundoscopy, and absence of concurring etiologies (particularly A-AION). Chances of reoccurrence of NAION in the affected eye are low; however, risk of NAION in the contralateral eye ranges between 12 and 25% over a 5-year period [2,4].

Beyond the management of modifiable risk factors, no established therapeutic or preventive measures are currently available for NAION [1,4,5,15,16,17]. Although a range of therapies have been explored, none have demonstrated a significant benefit in randomized clinical trials regarding vision improvement or prevention of contralateral eye involvement [5,16,17,18]. Acetylsalicylic acid has been evaluated for acute treatment or secondary prevention with no evidence of benefit compared to controls [19,20]. Similarly, corticosteroids, a crucial therapeutic measure in A-AION, have demonstrated no beneficial effect on visual outcome in NAION [21,22]. Surgical interventions like optic nerve sheath decompression (ONDS), vitrectomy or optic neurotomy have shown no clear benefit [23,24,25,26]. The Ischemic Optic Neuropathy Decompression Trial (IONDT) evaluated ONDS for NAION patients [23,24]. The trial was stopped prematurely, as visual improvement was lower and risk of further vision deterioration was statistically significantly higher in NAION patients undergoing ONDS compared to controls at 6-month follow-up [23,24]. Other explorative approaches, including neuroprotective and neuroregenerative therapies or intravitreal agents, have produced mixed results or are still being evaluated [5,16,17,18,27,28].

NAION results from reduced perfusion of the optic nerve head; however, its exact pathophysiology is not fully elucidated. It is generally considered a multifactorial disorder with various systemic and local factors contributing to its development [1,2,3,4,5]. The potential role of thromboembolism in the pathogenesis of NAION remains uncertain. Occasional case reports reported emboli as the presumed cause of NAION [29,30,31,32]. An early transcranial Doppler study did not reveal an increased microemboli rate in 11 patients with recent NAION compared to age-matched controls [33]. Evidence opposing an embolic cause includes the failure to detect a complete occlusion on fluorescein fundus angiography shortly after vison deterioration [1]. Interestingly, NAION was associated with a significantly higher risk of stroke, irrespective of comorbidities [34,35].

If an embolic origin—specifically, an occlusion of the short posterior ciliary arteries due to embolism of the ophthalmic artery—were to play a significant role in a subset of patients with NAION, one would expect concurrent embolic events in cerebral arteries. In patients with central retinal artery occlusion, a condition primarily considered embolic in nature [36], simultaneous ischemic cerebral infarctions were detected in up to one quarter of patients [37,38,39,40,41]. Given that both the central retinal artery and the posterior ciliary arteries originate from the ophthalmic artery, a shared embolic etiology would suggest that a proportion of NAION patients might also exhibit evidence of cerebral embolism.

Diffusion-weighted magnetic resonance imaging (DWI-MRI) is a well-established imaging sequence highly sensitive to microscopic changes of water diffusion in tissue. The corresponding apparent diffusion coefficient (ADC) provides a quantitative measure of the diffusion restriction. Together, these modalities enable precise and early detection of acute cerebral ischemia [42,43].

The purpose of this study was to assess the presence of concurrent cerebral infarctions in patients with NAION using DWI-MRI, thereby eliciting the possibility of a common embolic mechanism.

## 2. Materials and Methods

In this retrospective cohort study, cerebral DWI-MRI scans of patients with a new diagnosis of NAION were evaluated for concurrent acute or subacute ischemic stroke.

### 2.1. Patient Selection and Data Extraction

A medical database inquiry was performed to identify potential adult NAION patients with cerebral DWI-MRI treated at the Charité—Universitätsmedizin Berlin between 1 January 2013 and 31 December 2021. Patient records were identified using the International Classification of Diseases (ICD; H47.0—disorders of optic nerve, not elsewhere classified) and the German Operation and Procedure Classification System (OPS; 3-800—magnetic resonance imaging of cranium or 3-820—contrast-enhanced magnetic resonance imaging of cranium). NAION was considered present if the following criteria were met: (1) new onset of monocular or binocular non-traumatic, painless visual impairment; (2) evidence of optic disc edema on fundoscopy; (3) NAION documented as the diagnosis at discharge by the neuroophthalmologists; and (4) absence of other ocular, neurological, or systemic disorder that could better account for the symptoms. To exclusively examine diffusion restrictions that coincided with NAION, the duration between onset of visual impairment and time of cerebral DWI-MRI was limited to a maximum of 14 days.

Medical records were reviewed for relevant demographic, ophthalmological, and radiological data. The extent of vision impairment was classified according to the World Health Organization’s ICD (11th revision, 2025, Table 1).

To reduce uncertainty of a competing diagnosis of A-AION, records were additionally screened for information regarding giant cell arteritis based on the 2022 American College of Rheumatology/EULAR classification criteria for giant cell arteritis [44]. This included screening for clinical features (morning stiffness shoulders/arms or neck/torso, jaw or tongue claudication, new temporal headache, scalp tenderness, and abnormality of the A. temporalis superficialis), laboratory data (C-reactive protein, and erythrocyte sedimentation rate), evidence of hypoechoic wall thickening (“halo sign”) on ultrasound of the superficial temporal artery [45], and/or evidence of vasculitis via biopsy of the temporal artery or evidence of large-vessel vasculitis on computed tomography or magnetic resonance angiography.

This study was conducted in accordance with the Declaration of Helsinki and approved by the ethics committee of the Charité—Universitätsmedizin Berlin (registration number: EA1/235/23). Patient consent was waived by the ethics committee due to the retrospective design of the study.

### 2.2. Imaging Acquisition and Analysis

MR imaging was part of the routine diagnostic procedure of NAION patients. Images were acquired on two 1.5 T scanners (Magnetom Aera; Siemens, Erlangen, Germany) and one 3 T scanner (Magnetom Skyra; Siemens, Erlangen, Germany), each with a 20-channel head coil. At an additional 3 T scanner (Magnetom Trio; Siemens, Erlangen, Germany) with a 32-channel head coil, diffusion tensor imaging (DTI) was performed, which was used to calculate DWI and ADC values. Slice thicknesses ranged from 1.5 to 6.0 mm. MRI-DWI image analysis was conducted with the Merlin Diagnostic Workcenter (Phoenix-PACS GmbH, Freiburg, Germany). A board-certified neuroradiologist (E.S., >15 years of experience in MR imaging), blinded for patient diagnosis and clinical data, was presented pseudonymized DWI-MRI images. The images were evaluated for presence, location, and type of ischemic stroke. Evidence of ischemic stroke was defined as a demarcated hyperintense signal on DWI. A corresponding ADC hypointensity in the area of infarction enabled the differentiation between acute strokes (showing hypointense ADC values) and subacute strokes (with normal or elevated, but not reduced ADC signals). Patients were classified into two subgroups according to the duration between onset of vision impairment and time of DWI-MRI. DWI-MRI examinations performed within 0–6 days were defined as early and those performed on day 7–14 after visual impairment as late.

### 2.3. Statistical Analysis

A descriptive analysis was performed on the clinical data. Continuous variables were reported using measures of central tendency and variability (mean and standard deviation), while absolute and relative frequencies were used to summarize categorical variables. No inferential statistical testing was performed. All analyses were conducted within the R software environment (version 4.1.2, R Core Team) [46].

## 3. Results

**Figure 1:**
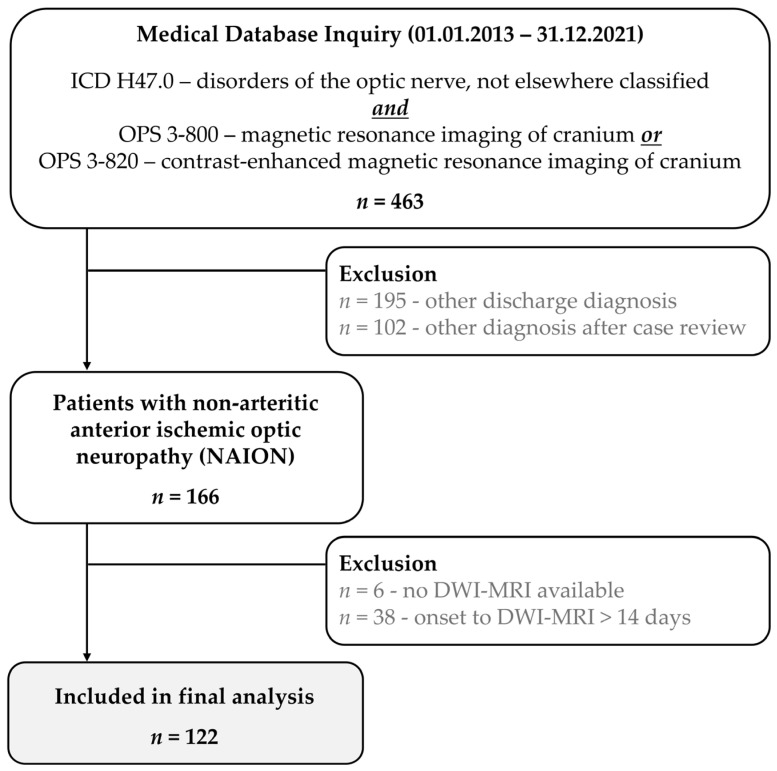
Patient selection process. ICD, International Classification of Diseases; OPS, Operation and Procedure Classification System; DWI-MRI, diffusion-weighted magnetic resonance imaging.

Medical database screening detected a total of 463 patient cases, which were reviewed for study inclusion. Of these, 195 were excluded based on ophthalmologic diagnosis at discharge and 102 cases after detailed review of available medical data. Of the remaining 166 cases that met our predetermined diagnostic criteria, 6 had to be excluded as no DWI sequences were acquired during MRI. In an additional 38 cases, the interval between onset of visual impairment and MRI exceeded 14 days. Ultimately, 122 NAION patients (44 female, M(SD) age = 64.6 (11.9) years) were included in the final study sample. The patient selection process is visualized in Figure 1.

The clinical and paraclinical characteristics of the cohort are listed in Table 2. NAION was left-sided in 73 patients (59.8%) and right-sided in 47 patients (38.5%). Two patients (1.6%) presented with bilateral NAION. In 51.6% of patients (*n* = 63) onset of visual impairment was sudden, whereas 12.3% (*n* = 15) described gradual visual impairment. Seventeen patients (13.9%) noticed visual impairment upon awakening. The overall quantitative visual acuity of the cohort was 0.4 ± 0.3 (decimal vision) or 0.40 ± 0.52 (logMAR). In the majority of patients (*n* = 58, 46.8%), visual acuity was classified as normal. Seven patients (5.6%) experienced mildly restricted visual acuity following NAION. Visual impairment was moderate in 30 patients (24.2%) and severe in 19 patients (15.3%). A total of 10 patients (8.1%) were classified as blind. RAPD in the eye affected by NAION was described in 62 patients (50.8%). In 30 patients (24.6%), it was not detected, while in further 17 patients (13.9%), it could not be evaluated due to prior pharmacological mydriasis required for the ophthalmological exam. In 11 patients (9.0%), RAPD was observed in the contralateral eye. All of these patients had a history of ophthalmological disorders (usually NAION) in that eye.

Diagnostic criteria of giant cell arteritis were only recorded in a fraction of the cohort during clinical work-up and are reported in Table 3. Overall, 8 of 91 patients (8.8%) in the NAION cohort reported a new temporal headache coinciding with the onset of visual impairment, and one patient of 34 (2.9%) reporting scalp tenderness. Morning stiffness or jaw or tongue claudication were not described in any patients. On clinical examination, abnormality of the temporal superficial artery was observed in one of 42 patients (2.4%). Laboratory examination showed an increased erythrocyte sedimentation rate (>50 mm/h) in 7 of 73 patients (11.3%) and an elevated C-reactive protein level (>10 mg/l) in 14 of 120 patients (11.7%). No patient demonstrated hypoechoic vessel wall thickening “halo sign” in ultrasonography. Temporal artery biopsy was performed in 11 patients (9.0%) and revealed no evidence of inflammatory activity. None of the patients fulfilled the criteria for giant cell arteritis [31].

Acquisition parameters of DWI-MRI are detailed in Table 4. In 63 patients (51.6%), DWI-MRI was performed within 0–6 days following onset of visual impairment. In 59 patients (48.4%), it was performed within 7–14 days.

Ischemic stroke was detected on DWI-MRI in three NAION patients (2.5%; one female). All lesions were located within the anterior circulation in the territory of the left middle cerebral artery. Ischemic stroke location was cortical in one case (0.8%) and subcortical in two cases (1.6%). Acute ischemic stroke indicated by a hyperintense DWI signal and corresponding low ADC was present in one patient (0.8%). This was the only patient with acute embolic stroke corresponding to the vascular territory of the affected eye. All strokes were clinically asymptomatic.

**Figure 2:**
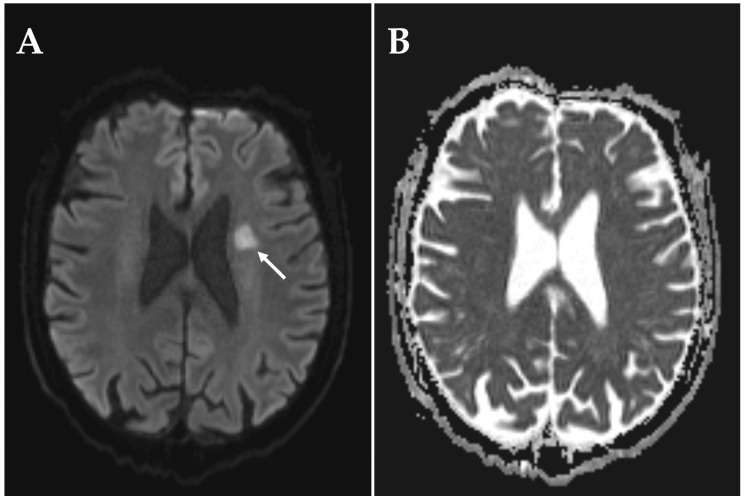
MRI sequences showing a subacute infarction in a patient with left-sided NAION. MRI was performed on a 3 T scanner within one day following onset of visual impairment. (**A**) DWI showing a subcortical hyperintense signal in the territory of the left middle cerebral artery. (**B**) ADC showing a normal signal in the corresponding region.

The first patient developed sudden vision impairment of the left eye in the middle of the day. The ophthalmologic exam revealed mild visual impairment (0.4) and a quadrant-surpassing, scotomatous mostly central visual field defect in the affected eye. RAPD was absent. The fundoscopic examination revealed temporal pallor of the optic disk. MRI-DWI performed one day after onset showed one subcortical hyperintense DWI lesion with a normal ADC, consistent with a subacute infarction which was considered to be primarily of microvascular and only secondarily of embolic origin (Figure 2). Further diagnostic work-up identified an ulcerous plaque in the left internal carotid artery without hemodynamically significant stenosis in duplex sonography. Transesophageal echocardiography revealed no sources of cardiac embolism.

**Figure 3:**
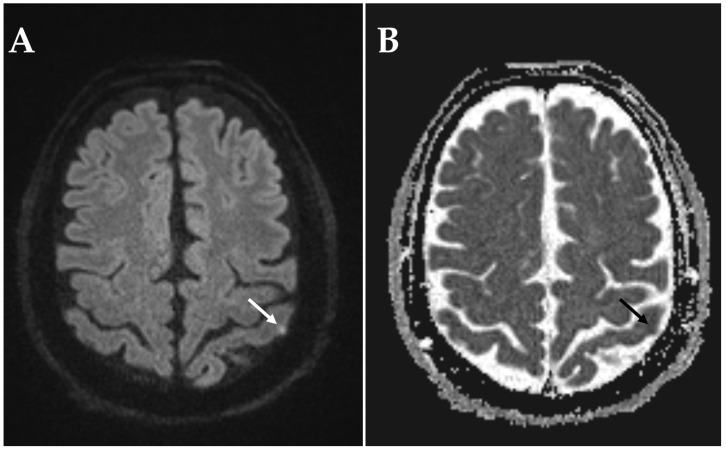
MRI sequences showing a small acute embolic infarction in a patient with NAION of the left-eye. MRI was performed on a 3 T scanner after three days following onset of visual impairment. (**A**) DWI reveals a small hyperintense cortical lesion in the territory of the left middle cerebral artery. (**B**) ADC with corresponding focal hypointense signal.

The second patient noticed a central visual field defect on the left eye upon awakening. With a quantitative visual acuity of 0.025, the patient was classified as blind on the affected eye. RAPD was present. The fundoscopic exam showed a pale, hyperemic optic disc with indistinct margins. MRI was performed three days after symptom onset and revealed a small cortical DWI hyperintensity deemed to be of embolic origin (Figure 3). Due to the small lesion size, ADC signal behavior was difficult to evaluate; however, it appeared to be mildly hypointense, consistent with an acute or early subacute ischemic stroke. Additionally, the MRI detected a chronic left posterior watershed and left anterior cerebral artery infarction. Duplex sonography excluded hemodynamically significant stenosis of the intra- and extracranial arteries. Transesophageal echocardiography revealed a persistent foramen ovale.

**Figure 4:**
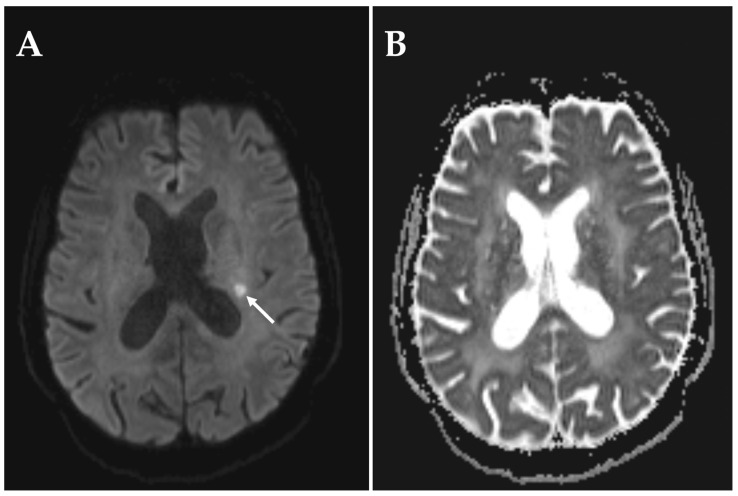
MRI sequences revealing subacute infarction in a patient with NAION of the right eye. MRI was performed on a 3 T scanner two days after onset of visual impairment. (**A**) DWI showing a hyperintensity in the subcortical territory of the left middle cerebral artery. (**B**) The corresponding region in the ADC shows normal signal behavior.

**Figure 5:**
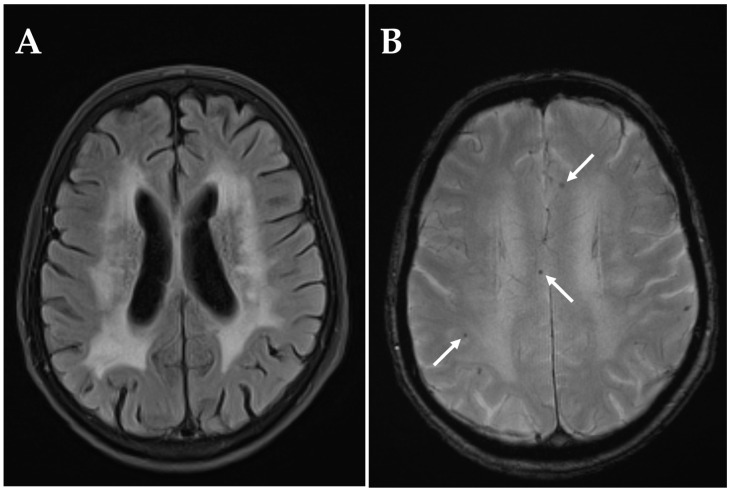
MRI indications of cerebral small vessel disease in a patient with NAION and presumably microvascular stroke. (**A**) Fluid-attenuated inversion recovery (FLAIR) sequence showing leukoaraiosis. (**B**) T2* sequence revealing several cortical microbleeds (arrows).

The third patient had a history of three ischemic strokes prior to onset of visual impairment (6 months prior: stroke in the territory of the left middle cerebral artery, one year prior: stroke in the territory of the right middle cerebral artery, and 3 years prior: stroke in the territory of the left middle cerebral artery). Under daily intake of acetylsalicylic acid 100 mg, the patient experienced a sudden central visual field defect in the right eye. Visual acuity was 0.04 and the patient was classified as blind in the affected eye. RAPD was present. The fundoscopic examination showed temporal pallor of the optic disc. An MRI performed two days following onset of visual symptoms revealed a subcortical DWI-hyperintensity with normal ADC signal (Figure 4). The stroke was classified as subacute and deemed to be of microvascular origin. This hypothesis was further supported by MRI findings compatible with cerebral small vessel disease, including leukoaraiosis and microbleeds among other features (Figure 5). No hemodynamically significant stenoses were found in duplex sonography of the intra- and extracranial arteries. A transesophageal echocardiography performed three years prior was normal and not repeated at time of NAION.

## 4. Discussion

The present study investigated the prevalence of concurrent acute or subacute cerebral infarctions in patients with newly diagnosed NAION. To this purpose, we performed a retrospective cohort study utilizing cerebral DWI-MRI scans performed within two weeks following visual impairment in patients with NAION, which were evaluated by a blinded, board-certified neuroradiologist for presence, location, and type of cerebral ischemia. Ischemic stroke was detected in a very small proportion of patients (3 of 122) and was located within the anterior circulation in the territory of the left middle cerebral artery in all cases. All strokes were clinically asymptomatic and concordantly small in size. In two cases, ischemic stroke was classified to be of microvascular origin, with one patient showing further radiographic evidence of cerebral small vessel disease (Figure 5). Only one NAION patient had an acute embolic stroke associated with the vascular territory of the impaired eye (Figure 3).

By comparison, central retinal artery and branch retinal arteriolar occlusion are considered embolic disorders of cardiac or large-vessel origin, causing acute ischemia of the retina [36]. Here, simultaneous ischemic strokes are found in up to a quarter of patients [37,38,39,40,41]. Both the central retinal artery afflicted in central retinal artery or branch retinal arteriolar occlusion and the posterior ciliary artery, which supplies the optic nerve head, originate from the ophthalmic artery. If thromboembolisms played a major role in the pathogenesis of NAION, a higher frequency of concomitant cerebral infarctions would be expected. It is reasonable to assume that the single case of acute embolic cerebral infarction in our cohort represents an incidental finding on DWI-MRI. Patients with NAION typically exhibit a cardiovascular risk profile that also predisposes them to systemic vascular disease, including ischemic stroke [34,35]. On the other hand, the two cases with microangiopathic ischemic infarctions did not represent acute cerebral infarcts. Thus, there was no strict temporal association with the occurrence of NAION. These lesions may instead reflect underlying cerebral small vessel disease, which has been reported to occur more frequently in patients with NAION [47]. In alignment with our results, emboli as the pathophysiological cause of NAION appear to be a rare occurrence [1,2,29,30,31,32,48].

Importantly, the prevalence of coincident ischemic stroke is much higher in A-AION [49]. A-AION is usually associated with giant cell arteritis and only seldomly described in other autoimmune or infectious vasculitis [1,2,4]. Giant cell arteritis is a CD4+T-cell-mediated autoimmune systemic vasculitis of the medium and large arteries, with a predilection for the posterior ciliary arteries [1,2,4]. Inflammation of the vessel wall causes thrombotic occlusion, which results in infarction of the optic nerve head and predisposes to ischemic stroke, particularly in the vertebrobasilar territory [1,2,4,49]. A complete occlusion of the posterior ciliary arteries is visible on fluorescein fundus angiography following onset of visual impairment [1,2]. Consequently, visual impairment in A-AION is usually more severe than in NAION [1,2,4].

Our understanding of the exact pathomechanism of NAION is still incomplete. Available evidence suggests a complex interplay of predisposing and precipitating factors, which compromise the circulation of the posterior ciliary artery and lead to a transient hypoperfusion or non-perfusion of the optic nerve head [1,2,3,4,5]. Predisposing systemic risk factors include, but are not limited to, diabetes mellitus, arterial hyper- and hypotension, sleep apnea, hyperlipidemia, and smoking [1,2,50,51]. A local risk factor is a small cup to disc ratio, also known as a “disc at risk” [4]. Further local predisposing risk factors include optic disc drusen and an increased intraocular pressure [1,2,3,4]. The acute vascular insufficiency is instigated by precipitating factors, particularly generalized or nocturnal arterial hypotension, which decrease the perfusion pressure of the optic nerve head below the critical autoregulatory level, resulting in NAION [1]. The ensuing hypoxia is accompanied by fluid extravasation and inflammatory processes leading to axonal damage of retinal ganglion cells [1,2,5]. Recent studies have highlighted a possible association between NAION and the glucagon-like peptide-1-receptor agonist semaglutide [52,53]. Although a direct mechanistic link has not yet been established, these findings have renewed interest in the underlying pathophysiology of NAION and may serve as a catalyst for future investigations that could ultimately prompt a revision of the current disease model [54].

Most characteristics of our NAION cohort were consistent with commonly described clinical features of the disorder. Consistent with the literature, NAION was more prevalent in men and affected more older than younger patients in our cohort [1,2,5]. The patients in this cohort displayed a wide range of visual impairment; most patients were classified as having no relevant vision impairment according to the World Health Organization’s ICD. Interestingly, in two of 122 patients, bilateral NAION was described. According to Hayreh, simultaneous bilateral NAION is exceedingly rare and only described in cases of extreme sudden hypotension [1,2]. In our cohort, we found no evidence of extreme hypotension and both patients only experienced vision loss in one eye. Affection of the second eye was solely detected on fundoscopic examination. It is likely that in these cases, NAION occurred in two stages and not simultaneously. The onset of visual impairment in our cohort is compatible with the hypothesized pathophysiology described above. Most patients described sudden visual loss, while a significant proportion noticed symptoms upon awakening, possibly following an episode of nocturnal hypotension. In NAION, progressive worsening is not uncommon and presumed to correlate with a local compartment syndrome due to the optic disc edema causing progressive ischemia [4].

The present study has several limitations owed to its retrospective design. The process of patient selection using ICD and OPS codes may have missed NAION patients if these were misdiagnosed or misclassified. As the reviewed data was not originally collected for research, the compiled data (e.g., clinical patient characteristics) was partially incomplete. The DWI sequences were part of the routine diagnostic work-up of NAION patients and were acquired on four separate MRI scanners with varying head coils, field strengths, and slice thicknesses. However, we used established MRI sequences commonly applied in clinical practice and MRI has a high sensitivity for detecting acute cerebral ischemia independent of the used protocol [42,43,55,56]. In our study, image analysis was performed by a single rater. However, due to the high accuracy in lesion detection, DWI provides good interrater reliability and thus rating by a second reader was not deemed necessary [56]. We ascertained blinding for clinical data and patient diagnosis of the reader to mitigate a potential detection bias. Lastly, it is important to note that lacunar-appearing infarcts on DWI have been observed in patients with atrial fibrillation and are therefore not exclusively attributable to cerebral small vessel disease—particularly when presenting as an isolated, acute, small deep infarction without accompanying white matter disease or chronic lacunar lesions [57,58]. Accordingly, increased detection rates of atrial fibrillation have been reported among patients with suspected small-vessel occlusive disease [59]. A clear strength of the study is the careful verification of NAION diagnosis in the cohort after detailed individual case review.

## 5. Conclusions

In this retrospective cohort of 122 patients with newly diagnosed NAION who underwent DWI-MRI within 14 days of visual impairment, concurrent cerebral ischemia was uncommon, with only three cases (2.5%) identified. Of these, only one represented an acute embolic infarct anatomically corresponding to the vascular territory of the affected eye. The remaining two lesions displayed imaging characteristics suggestive of small-vessel occlusion, consistent with prior reports of a higher burden of cerebral small vessel disease in patients with NAION. To our knowledge, this is the first study to investigate the frequency of acute ischemic stroke in NAION using DWI-MRI. By restricting MRI acquisition to the first 14 days after visual impairment and differentiating acute from subacute lesions using combined DWI/ADC signal characteristics, our study provides a precise estimate of coincident cerebral ischemia in NAION. A methodological strength of this study is the stringent exclusion of A-AION patients based on detailed chart review and current diagnostic criteria for giant cell arteritis. Taken together, our study establishes a realistic baseline prevalence of incidental acute ischemic lesions in NAION and provides additional evidence that embolism is unlikely to meaningfully contribute to NAION pathophysiology.

## Figures and Tables

**Table 1:** Levels of vision impairment according to the World Health Organizations International Classification of Diseases.

Category	Visual Acuity
No vision impairment	≥6/12 (≤0.30 logMAR)
Mild vision impairment	<6/12–≥6/18 (>0.30–≤0.48 logMAR)
Moderate vision impairment	<6/18–≥6/60 (>0.48–≤1.00 logMAR)
Severe vision impairment	<6/60–≥3/60 (>1.00–≤1.30 logMAR)
Blindness	<3/60 (>1.30 logMAR)

**Table 2:** NAION patient characteristics.

Demographic Information	NAION Cohort (*n* = 122)
Age (years, mean ± SD)	64.6 ± 11.9
Female (*n*, %)	44 (36.1)
**Ophthalmological features**	
NAION left eye (*n*, %)	73 (59.8)
NAION right eye (*n*, %)	47 (38.5)
Bilateral NAION (*n*, %)	2 (1.6)
**Onset**	
Sudden (*n*, %)	63 (51.6)
Gradual (*n*, %)	15 (12.3)
Upon awakening (*n*, %)	17 (13.9)
Not reported (*n*, %)	27 (22.1)
Visual Impairment *	
Quantitative decimal visual acuity (mean ± SD)	0.4 ± 0.3
Quantitative logMAR visual acuity (mean ± SD)	0.40 ± 0.52
No vision impairment (*n*, %)	58 (46.8)
Mild (*n*, %)	7 (5.6)
Moderate (*n*, %)	30 (24.2)
Severe (*n*, %)	19 (15.3)
Blind (*n*, %)	10 (8.1)
**RAPD**	
Present on affected eye (*n*, %)	62 (50.8)
Not present (*n*, %)	30 (24.6)
Present in other eye (*n*, %)	11 (9.0)
Pharmacological mydriasis (*n*, %)	17 (13.9)
Not reported (*n*, %)	2 (1.6)

NAION, non-arteritic anterior ischemic optic neuropathy; RAPD, relative afferent pupillary defect; * visual impairment categorized based on the World Health Organization’s International Classification of Diseases (ICD, 11th revision, 2025).

**Table 3:** NAION patient characteristics regarding giant cell arteritis diagnostic criteria.

Classification Criteria for Giant Cell Arteritis	NAION Cohort (*n* = 122)
Morning stiffness shoulders/arms, neck/torso (*n*, %)	0/23 (0)
Jaw or tongue claudication (*n*, %)	0/77 (0)
New temporal headache (*n*, %)	8/91 (8.8)
Scalp tenderness (*n*, %)	1/34 (2.9)
Abnormality of the A. temporalis superficialis (*n*, %)	1/42 (2.4)
ESR > 50 mm/h (*n*, %)	7/73 (11.3)
ESR (mm/h, mean ± SD) ˠ	18.3 ± 16.7
C-reactive protein > 10 mg/l (*n*, %)	14/120 (11.7)
C-reactive protein (mg/l, mean ± SD) ˣ	8.9 ± 35.3
“Halo-sign” present (*n*, %)	0/66 (0)
Temporal artery biopsy positive (*n*, %)	0/11 (0)
Radiological evidence of large-vessel vasculitis	0/0 (0)

NAION, non-arteritic anterior ischemic optic neuropathy; ESR, erythrocyte sedimentation rate; ˠ measured and documented in 62 cases; ˣ measured and documented in 120 cases.

**Table 4:** DWI-MRI acquisition parameters.

	Total (*n* = 122)	Early DWI-MRI Day 0–6 (*n* = 63) ˠ	Late DWI-MRI Day 7–14 (*n* = 59) ˣ
**Field strength (Tesla)**			
1.5 (*n*, %)	67 (54.9)	29 (46.0)	38 (64.4)
3.0 (*n*, %)	55 (45.1)	34 (54.0)	21 (35.6)
**Slice thickness (mm)**			
1.5 (*n*, %)	2 (1.6)	1 (1.6)	1 (1.7)
2.0 (*n*, %)	1 (0.8)	0	1 (1.7)
2.5 (*n*, %)	32 (26.2)	19 (30.2)	13 (22.0)
3.0 (*n*, %)	80 (65.6)	40 (63.5)	40 (67.8)
5.0 (*n*, %)	6 (4.9)	2 (3.2)	4 (6.8)
6.0 (*n*, %)	1 (0.8)	1 (1.6)	0

DWI-MRI, diffusion-weighted magnetic resonance imaging; ˠ *n* = 3 cases where exact date of onset is unclear; ˣ *n* = 13 cases where exact date of onset is unclear.

## Data Availability

The data presented in this study is available on request from the corresponding author. The data are not publicly available due to ethical and privacy restrictions.

## Abbreviations

The following abbreviations are used in this manuscript:

ION	Ischemic optic neuropathies
AION	Anterior ischemic optic neuropathy
PION	Posterior ischemic optic neuropathy
NAION	Non-arteritic anterior ischemic optic neuropathy
A-AION	Arteritic anterior ischemic optic neuropathy
RAPD	Relative afferent pupillary defect
ONDS	Optic nerve sheath decompression
DWI-MRI	Diffusion-weighted magnetic resonance imaging
ADC	Apparent diffusion coefficient
ICD	International Classification of Diseases
OPS	Operation and Procedure Classification System
FLAIR	Fluid-attenuated inversion recovery
